# Interaction of the Effects of Alcohol Drinking and Polymorphisms in Alcohol-Metabolizing Enzymes on the Risk of Female Breast Cancer in Japan

**DOI:** 10.2188/jea.JE20081035

**Published:** 2009-09-05

**Authors:** Takakazu Kawase, Keitaro Matsuo, Akio Hiraki, Takeshi Suzuki, Miki Watanabe, Hiroji Iwata, Hideo Tanaka, Kazuo Tajima

**Affiliations:** 1 Division of Epidemiology and Prevention, Aichi Cancer Center Research Institute, Nagoya, Japan; 2 Department of Breast Oncology, Aichi Cancer Center Hospital, Nagoya, Japan; 3 Department of Epidemiology, Nagoya University Graduate School of Medicine, Nagoya, Japan

**Keywords:** breast cancer, alcohol drinking, acetaldehyde, polymorphisms in alcohol-metabolizing enzyme genes, case–control study

## Abstract

**Background:**

Epidemiological studies consistently indicate that alcoholic beverages are an independent risk factor for female breast cancer. Although the mechanism underlying this effect remains unknown, the predominant hypothesis implicates mutagenesis via the ethanol metabolite acetaldehyde, whose impact on the carcinogenesis of several types of cancer has been shown in both experimental models and molecular epidemiological studies. Many of the epidemiological studies have investigated genetic polymorphisms of alcohol dehydrogenase-1B (ADH1B) His48Arg and aldehyde dehydrogenase-2 (ALDH2) Glu504Lys, because of the strong impact these polymorphisms have on exposure to and accumulation of acetaldehyde. With regard to breast cancer, however, evidence is scarce.

**Methods:**

To clarify the impact on female breast cancer risk of the interaction of the effects of alcohol consumption and polymorphisms in the alcohol-metabolizing enzymes *ADH1B* and *ALDH2*, we conducted a case–control study of 456 newly and histologically diagnosed breast cancer cases and 912 age- and menopausal status-matched noncancer controls. Gene–gene and gene–environment interactions between individual and combined *ADH1B* and *ALDH2* gene polymorphisms and alcohol consumption were evaluated.

**Results:**

Despite sufficient statistical power, there was no significant impact of *ADH1B* and *ALDH2* on the risk of breast cancer. Neither was there any significant gene–environment interactions between alcohol drinking and polymorphisms in *ADH1B* and *ALDH2*.

**Conclusions:**

Our findings do not support the hypothesis that acetaldehyde is the main contributor to the carcinogenesis of alcohol-induced breast cancer.

## INTRODUCTION

Breast cancer now ranks first among cancers that affect women worldwide.^1^ Numerous epidemiological studies have indicated that alcoholic beverages are an independent risk factor for female breast cancer.^2^^,^^3^ Recently, the International Agency for Research on Cancer (IARC) noted that there is sufficient evidence to classify alcohol as a carcinogen in the development of human female breast cancer.^4^ However, the mechanism responsible for breast carcinogenesis due to consumption of alcoholic beverages remains unknown.^5^ One hypothesis suggests that mutagenesis is caused by the ethanol metabolite acetaldehyde. Indeed, the carcinogenic effect of acetaldehyde has been clearly shown in experimental models of several types of cancer^6^^–^^8^ and its impact on human cancer risk has been confirmed in molecular epidemiological studies for several cancers,^9^^–^^15^ Nevertheless, evidence with regard to breast cancer is scarce.^16^

Polymorphisms in alcohol-metabolizing enzyme genes cause marked variation in acetaldehyde concentrations among individuals. Ethanol is oxidized to acetaldehyde by the alcohol dehydrogenase enzymes (ADH), particularly ADH1B. Acetaldehyde is then further oxidized and detoxified into acetate by aldehyde dehydrogenases (ALDHs), and this oxidation owes much to ALDH2. Genes that encode these 2 representative alcohol-metabolizing enzymes display polymorphisms that modulate individual differences in alcohol-detoxifying capability and drinking behavior.^17^ Regarding the *ADH1B* Arg48(*1:slow)/His(*2:rapid) polymorphism, the ADH1B*2 allele is a superactive subunit of ADH1B that confers an approximately 40-times-higher Vmax than the less active ADH1B*1/*1 form.^18^^,^^19^ In contrast, for the *ALDH2* Glu504(*1:active)/Lys504(*2:null) polymorphism, the ALDH2*2 allele encodes a catalytically inactive subunit.^18^^,^^19^ Individuals with the ALDH2*1/*2 genotype have only 6.25% of the normal level of ALDH2 protein. The ADH1B*1 and ALDH2*2 alleles, which are associated with prolonged exposure to or higher accumulation of acetaldehyde, are clustered in east Asian populations,^20^^,^^21^ including Japanese, and lead to high acetaldehyde concentrations upon alcohol consumption in affected individuals.

Given this background, molecular epidemiological studies have investigated the carcinogenic impact of acetaldehyde by examining possible gene–environment interactions of the effects of alcohol consumption and polymorphisms in alcohol-metabolizing enzyme genes on the risk of several types of cancer. The results revealed effect modifications by these polymorphisms for several types of cancer in individuals expected to be rapidly or extensively exposed to acetaldehyde due to the presence of *ADH1B* and/or *ALDH2* gene polymorphisms.^9^^–^^14^

We conducted a case–control study to determine the impact of the interaction of the effects of alcohol consumption and polymorphisms in the alcohol-metabolizing enzymes ADH1B and ALDH2 on the risk of female breast cancer in Japan.

## METHODS

### Subjects

The cases were 456 patients with no previous history of cancer who were newly and histologically diagnosed with breast cancer from January 2001 through June 2005 at Aichi Cancer Center Hospital in Japan. The controls were 912 subjects who were randomly selected and matched by age (±3 years) and menopausal status (pre- or postmenopause) to cases in a 1:2 case–control ratio. This sample size was selected to enable the detection of gene–environment interactions between *ALDH2* and alcohol consumption, after calculating the odds ratio (OR) for ALDH2*2 (1.2, log-additive), the OR for alcohol drinking (1.2, binary), and the interaction OR between them (2.0) that provided greater than 95% power. All subjects were recruited within the framework of the Hospital-based Epidemiologic Research Program at Aichi Cancer Center (HERPACC), as described elsewhere.^22^^,^^23^ In brief, information on lifestyle factors was collected using a self-administered questionnaire and checked by a trained interviewer. Outpatients were also asked to provide blood samples. Each patient was asked about his or her lifestyle when healthy or before the current symptoms developed. Approximately 95% of eligible subjects completed the questionnaire and 60% provided blood samples. We used noncancer patients at our hospital as controls, given the high likelihood that our cases were members of this population. We previously confirmed the feasibility of using noncancer outpatients at our hospital as controls in epidemiological studies by showing that their general lifestyles accorded with those of a general population randomly selected from the electoral roll in Nagoya City, Aichi Prefecture.^24^ The data were loaded into a HERPACC database and routinely linked with the hospital-based cancer registry system to update the data on cancer incidence. All participants gave written informed consent and the study was approved by Ethical Committee of Aichi Cancer Center.

### Genotyping of ADH1B and ALDH2

DNA from each subject was extracted from the buffy coat fraction with a BioRobot EZ1 with an EZ1 DNA Blood 350-µL kit or QIAamp DNA Blood Mini Kit (Qiagen K. K., Tokyo, Japan). Genotyping was based on Taqman Assays from Applied Biosystems (Foster City, CA, USA). The principle of the TaqMan Real-Time polymerase chain reaction (PCR) assay system using fluorogenic probes and 5' nuclease has been described by Livak.^25^ All of the assays were done in 96-well PCR plates. Amplification reactions (5 µL) were done in duplicate with 30 ng of template DNA, 2× TaqMan Universal Master Mix buffer (Applied Biosystems), 20× primer, and probe mix (Applied Biosystems). Thermal cycling was initiated with a first denaturation step of 20 seconds at 95 °C, and then by 40 cycles of 3 seconds at 95 °C and 30 seconds at 62 °C. After PCR was completed, plates were brought to room temperature and read using a 7500 Fast Real-Time PCR System (Applied Biosystems), and the results were analyzed using the 7500 Fast System SDS software. The quality of genotyping in our department is routinely assessed by conducting re-genotyping of a randomly selected 5% of samples, and complete concordance of the results of the genotyping was confirmed. We also confirmed that there were no allelic distributions among the controls that departed from the Hardy–Weinberg frequency.

### Alcohol consumption and other environmental exposures

Consumption of each type of beverage (Japanese *sake*, beer, *shochu*, whiskey, and wine) was defined as the average number of drinks per day, which was then converted into a Japanese *sake* (rice wine) equivalent. One drink is equal to 1 *go* (180 mL) of Japanese *sake*, which contains 23 g of ethanol and is equivalent to 1 large bottle (633 mL) of beer, 2 shots (60 mL) of whiskey, or 2.5 glasses of wine (200 mL). One drink of *shochu* (distilled spirit), which contains 25% ethanol, was rated as 108 mL. Total alcohol consumption was estimated as the amount of pure alcohol consumption (g/drink) of Japanese *sake*, beer, *shochu*, whiskey, and wine among current regular drinkers. Alcohol consumption was classified into 5 categories: never, former, and—for current drinkers—light, moderate, and heavy. Heavy drinkers were defined as those currently consuming at least 15 g of pure alcohol per day, moderate drinkers as those currently consuming between 5 g and less than 15 g per day, and light drinkers as those currently consuming less than 5 g per day. Cumulative smoking dose was evaluated in pack-years, ie, the product of the number of packs consumed per day and years of smoking. Smoking status was classified into 4 categories: never, former, current smoker of <20 pack-years, and current smoker of ≥20 pack-years. Former drinkers and former smokers were defined as those who had quit drinking or smoking at least 1 year before the survey, respectively. Body-mass index (BMI) was calculated as weight divided by height squared (kg/m^2^). Regular exercise was defined as exercise activity more than once per month, regardless of exercise time. In our study, family history was considered positive if either a mother or sister had had breast cancer.

### Statistical analysis

To assess the strength of the associations between polymorphic genes involved in ADH1B and ALDH2 metabolism and the risk of breast cancer, odds ratios (ORs) with 95% confidence intervals (CIs) were estimated using logistic regression models adjusted for potential confounders. Although we attempted conditional logistic regression as primary analysis, we finally applied unconditional logistic regression to avoid the dropping of controls, which led to unstable estimation in stratified analysis. Consistency between conditional and unconditional logistic regression models was confirmed. Potential confounders considered in the multivariate analyses were age (as a continuous variable), alcohol consumption (never, former, light, moderate, heavy), smoking status (never, former, current smokers of <20 or ≥20 pack-years), current BMI (<18.5, 18.5–24.9, ≥25.0), regular exercise (yes, no), family history of breast cancer (yes, no), menopausal status (premenopause, postmenopause), age at menarche (≤12, 13–14, ≥15 years), parity (0, 1–2, ≥3), past use of hormone-replacement therapy (never, 1–6 months, >6 months), and mode of referral to our hospital (patient’s discretion, family or friend’s recommendation, referral from another clinic, secondary screening after primary screening, or other). To account for possible differences between cases and controls, we adjusted for mode of referral to our hospital. Differences in categorized demographic variables between the cases and controls were tested using the chi-square test. Mean ages of cases and controls were compared using the *t*-test. To assess for discrepancies between genotype and allele frequencies, accordance with the Hardy-Weinberg equilibrium was checked for controls, using the chi-square test. As a basis for the trend test, we assigned scores for genotype (0, homozygous genotype for reference allele; 1, heterozygous genotype; and 2, homozygous genotype for nonreference allele) and alcohol consumption (0, never-drinker; 1, former drinker; 2, light drinker; 3, moderate drinker; and 4, heavy drinker), which were then included in the model. In interaction analysis, products of scores for genotype and alcohol consumption, as described above, were included as interaction terms. A *P* value less than 0.05 was considered statistically significant. All analyses were performed using STATA version 10 (Stata Corp., College Station, TX, USA), except for the power calculations, which were performed using QUANTO version 1.2.^26^

## RESULTS

Data from 456 breast cancer cases and 912 controls were available for analysis. Table 1 shows the distribution by background characteristics. Age was appropriately matched, and drinking and smoking habits did not significantly differ between groups. With regard to mode of referral, family recommendation and referral from other clinics were more frequent among the cases than among controls; patient’s discretion and secondary screening were less frequent among cases.

**Table 1.** Characteristics of cases and controls

	Cases (*n* = 456), *n* (%)	Controls (*n* = 912), *n* (%)	*P*
Age (years)			
​ ≤29	5 (1.1)	10 (1.1)	
​ 30–39	46 (10.1)	76 (8.3)	
​ 40–49	125 (27.4)	259 (28.4)	
​ 50–59	148 (32.5)	285 (31.3)	
​ 60–69	101 (22.1)	205 (22.5)	
​ 70–79	31 (6.8)	77 (8.4)	0.80
​ Mean age (SD)	52.8 (10.7)	53.6 (10.8)	0.25

Alcohol consumption			
​ Never	286 (61.7)	563 (61.7)	
​ Former^a^	8 (1.8)	15 (1.6)	
​ Current			
​ ​ Light (<5 g ethanol/day)	73 (16.0)	157 (17.2)	
​ ​ Moderate (≥5 g and ​ ​ ​ <15 g ethanol/day)	50 (11.0)	103 (11.3)	
​ ​ Heavy (≥15 g ethanol/day)	36 (7.9)	59 (6.5)	0.88
​ Unknown	3 (0.7)	15 (1.6)	

Smoking status			
​ Never	382 (83.8)	724 (79.4)	
​ Former^c^	24 (5.3)	55 (6.0)	
​ Current (pack years)			
​ ​ 0–19	34 (7.5)	78 (8.6)	
​ ​ ≥20	14 (3.1)	53 (5.8)	0.11
​ Unknown	2 (0.4)	2 (0.2)	

BMI			
​ <18.5	37 (8.1)	70 (7.7)	
​ 18.5–24.9	324 (71.1)	671 (73.6)	
​ ≥25.0	95 (20.8)	166 (18.2)	0.49
​ Unknown	0 (0)	5 (0.5)	

Regular exercise			
​ Yes	297 (65.1)	623 (68.3)	
​ No	157 (34.4)	288 (31.6)	0.27
​ Unknown	2 (0.4)	1 (0.1)	

Family history of breast cancer			
​ Yes	32 (7.0)	47 (5.2)	
​ No	399 (87.5)	781 (85.6)	0.23
​ Unknown	25 (5.5)	84 (9.2)	

Menopausal status			
​ Premenopausal	217 (47.6)	434 (47.6)	
​ Postmenopausal	239 (52.4)	478 (52.4)	1.00

Age at menarche (years)			
​ ≤12	132 (28.9)	239 (26.2)	
​ 13–14	218 (47.8)	443 (48.6)	
​ ≥15	103 (22.6)	207 (22.7)	0.68
​ Unknown	3 (0.7)	23 (2.5)	

Age at menopause (years)			
​ ≤47	53 (22.2)	103 (21.5)	
​ 48–52	121 (50.6)	253 (52.9)	
​ ≥53	64 (26.8)	116 (24.3)	0.53
​ Unknown	1 (0.4)	6 (1.3)	

Parity			
​ 0	61 (13.4)	133 (14.6)	
​ 1–2	300 (65.8)	541 (59.3)	
​ 3	95 (20.8)	232 (25.4)	0.08
​ Unknown	0 (0)	6 (0.7)	

Hormone replacement therapy (months)			
​ Never	395 (86.6)	755 (82.8)	
​ 1–6	32 (7.0)	80 (8.8)	
​ >6	27 (5.9)	64 (7.0)	0.34
​ Unknown	2 (0.4)	13 (1.4)	

Mode of referral to hospital			
​ Patient’s discretion	124 (27.2)	287 (31.5)	
​ Family recommendation	114 (25.0)	153 (16.8)	
​ Referral from other clinics	130 (28.5)	177 (19.4)	
​ Secondary screening after ​ ​ primary screening	84 (18.4)	286 (31.4)	
​ Other	2 (0.4)	6 (0.7)	<0.01
​ Unknown	2 (0.4)	3 (0.3)	

SD: standard deviation, BMI: body-mass index.

^a^Former smokers and former drinkers were defined as subjects who had quit smoking or drinking at least 1 year before completing the questionnaire.

Table 2 shows genotype distributions for *ADH1B* and *ALDH2*, and individual ORs and 95% CIs for breast cancer risk. Genotype frequencies for both polymorphisms were in accordance with the Hardy–Weinberg law in controls: *ADH1B* His48Arg (*P* = 0.90) and *ALDH2* Glu504Lys (*P* = 0.10). No significant impact on breast cancer risk by genotype was seen with individual polymorphisms for either *ADH1B* or *ALDH2* in the analysis controlling for matching factors only or the analysis also controlling for potential confounders.

**Table 2.** Risk of female breast cancer, by ADH1B and ALDH2 genotype

	Cases /Controls	OR-1^a^ (95% CI)	OR-2^b^ (95% CI)
ADH1B			
​ (*2:rapid/*2:rapid)	265/539	1 (ref.)	1 (ref.)
​ (*1:slow/*2:rapid)	162/322	1.02 (0.81–1.3)	1.05 (0.82–1.34)
​ (*1:slow/*1:slow)	25/47	1.08 (0.65–1.79)	1.15 (0.69–1.94)
​ Unknown	4/4		
​ *P*_trend_^c^		0.75	0.541

ALDH2			
​ (*1:active/*1:active)	222/455	1 (ref.)	1 (ref.)
​ (*1:active/*2:null)	196/362	1.11 (0.88–1.41)	1.08 (0.83–1.39)
​ (*2:null/*2:null)	38/93	0.83 (0.55–1.25)	0.77 (0.49–1.19)
​ Unknown	0/2		
​ *P*_trend_^c^		0.846	0.568

^a^Logistic regression model controlling for matching factors only.

^b^Logistic regression model controlling for matching factors plus alcohol consumption, smoking status, body-mass index, regular exercise, family history of breast cancer, age at menarche, parity, hormone-replacement therapy, and mode of referral to hospital.

^c^Trends for genotypes were assessed by a test that used scores for each genotype (0, homozygous genotype for reference allele; 1, heterozygous genotype; and 2, homozygous genotype for nonreference allele).

Table 3 shows the impact of alcohol consumption overall and by individual *ADH1B* and *ALDH2* genotype. In overall analysis, heavy drinkers (those consuming more than 15 g ethanol/day) versus nondrinkers, the point estimate of the OR was greater than unity (OR, 1.33; 95% CI, 0.84 to 2.11; *P* = 0.230), although not significantly so. Former drinkers with ADH1B*1/*1 and all categories with ALDH2*2/*2 could not be analyzed because of the small numbers of case and control subjects. None of the available analyses stratified by individual *ADH1B* and *ALDH2* genotypes showed a significant association with alcohol drinking status.

**Table 3.** Impact of alcohol consumption on the risk of female breast cancer, by ALDH2/ADH1B genotype

	Cases /Controls	OR-1^a^ (95% CI)	OR-2^b^ (95% CI)
All			
​ Never-drinker	286/563	1 (ref.)	1 (ref.)
​ Former drinker	8/15	1.03 (0.43–2.47)	1.17 (0.48–2.83)
​ Light drinker	73/157	0.9 (0.66–1.23)	0.92 (0.67–1.26)
​ Moderate drinker	50/103	0.93 (0.64–1.35)	0.95 (0.65–1.39)
​ Heavy drinker	36/59	1.17 (0.76–1.82)	1.33 (0.84–2.11)
​ Unknown	3/15		
​ *P*_trend_^c^		0.981	0.72

ADH1B (*2:rapid/*2:rapid)			
​ Never-drinker	165/337	1 (ref.)	1 (ref.)
​ Former drinker	6/9	1.36 (0.48–3.89)	1.78 (0.59–5.34)
​ Light drinker	40/90	0.91 (0.6–1.38)	0.93 (0.61–1.43)
​ Moderate drinker	31/65	0.97 (0.61–1.55)	1.06 (0.66–1.72)
​ Heavy drinker	20/33	1.24 (0.69–2.22)	1.61 (0.85–3.02)
​ Unknown	3/5		
​ *P*_trend_^c^		0.829	0.418

ADH1B (*1:slow/*2:rapid)			
​ Never-drinker	104/200	1 (ref.)	1 (ref.)
​ Former drinker	2/4	0.97 (0.17–5.39)	0.91 (0.16–5.11)
​ Light drinker	26/56	0.88 (0.52–1.49)	0.85 (0.5–1.45)
​ Moderate drinker	17/33	0.97 (0.51–1.85)	0.88 (0.45–1.71)
​ Heavy drinker	13/21	1.17 (0.56–2.45)	1.12 (0.52–2.4)
​ Unknown	0/8		
​ *P*_trend_^c^		0.956	0.832

ADH1B (*1:slow/*1:slow)			
​ Never-drinker	15/23	1 (ref.)	1 (ref.)
​ Former drinker	0/2	NA NA	NA NA
​ Light drinker	5/10	0.73 (0.2–2.6)	0.82 (0.21–3.25)
​ Moderate drinker	2/5	0.65 (0.11–3.92)	0.59 (0.09–3.93)
​ Heavy drinker	3/5	1 (0.2–4.92)	1.7 (0.16–17.69)
​ Unknown	0/2		
​ *P*_trend_^c^		0.764	0.887

ALDH2 (*1:active/*1:active)			
​ Never-drinker	99/196	1 (ref.)	1 (ref.)
​ Former drinker	5/11	0.91 (0.31–2.7)	1.03 (0.34–3.16)
​ Light drinker	47/108	0.86 (0.56–1.32)	0.88 (0.57–1.36)
​ Moderate drinker	37/75	0.99 (0.62–1.58)	0.98 (0.61–1.59)
​ Heavy drinker	31/53	1.17 (0.7–1.95)	1.21 (0.7–2.11)
​ Unknown	3/12		
​ *P*_trend_^c^		0.801	0.887

ALDH2 (*1:active/*2:null)			
​ Never-drinker	149/273	1 (ref.)	1 (ref.)
​ Former drinker	3/4	1.34 (0.29–6.11)	1.94 (0.4–9.27)
​ Light drinker	26/49	0.93 (0.55–1.57)	0.97 (0.57–1.67)
​ Moderate drinker	13/27	0.82 (0.4–1.66)	0.85 (0.41–1.76)
​ Heavy drinker	5/6	1.51 (0.45–5.07)	1.82 (0.52–6.36)
​ Unknown	0/3		
​ *P*_trend_^c^		0.871	0.892

ALDH2 (*2:null/*2:null)			
​ Never-drinker	38/92	1 (ref.)	1 (ref.)
​ Former drinker	0/0	NA NA	NA NA
​ Light drinker	0/0	NA NA	NA NA
​ Moderate drinker	0/1	NA NA	NA NA
​ Heavy drinker	0/0		
​ Unknown	0/0		
​ *P*_trend_^c^		NA	NA

^a^Logistic regression model controlling for matching factors only.

^b^Logistic regression model controlling for matching factors plus alcohol consumption, smoking status, body-mass index, regular exercise, family history of breast cancer, age at menarche, parity, hormone-replacement therapy, and mode of referral to hospital.

^c^Trends for genotypes were assessed by a test that used scores for drinking status (0, Never-drinker; 1, Former drinker; 2, Light drinker; 3, Moderate drinker and 4, Heavy drinker).

Table 4 shows the impact of the combined *ADH1B* and *ALDH2* genotypes. Among all 9 genotype combinations, only the combination of ADH1B*1/*2 and ALDH2*2/*2 was significantly associated with a reduced risk of breast cancer, although it should be mentioned that all subjects with this genotype combination were never-drinkers. None of the available analyses for the combined genotype showed a significant association with increased or reduced risk (data not shown) for any type of drinker (former, light, moderate, or heavy), although genotype combinations which included ALDH2*2/*2 could not be analyzed because of the small number of drinkers with the ALDH2*2/*2 genotype (see Table 3).

**Table 4.** Effect of combined ADH1B and ALDH2 genotype on the risk of female breast cancer

	ADH1B (*2:rapid/*2:rapid)			ADH1B (*1:slow/*2:rapid)			ADH1B (*1:slow/*1:slow)		
		
	Cases /Controls	OR (95% CI)	*P*	Cases /Controls	OR (95% CI)	*P*	Cases /Controls	OR (95% CI)	*P*
ALDH2 (*1:active/*1:active)	132/278			77/148			9/24		
​ OR-1^a ^(95% CI)		1 (ref.)			1.1 (0.78–1.55)	0.589		0.79 (0.36–1.75)	0.559
​ OR-2^b ^(95% CI)		1 (ref.)			1.13 (0.8–1.61)	0.483		0.84 (0.37–1.89)	0.675
									
ALDH2 (*1:active/*2:null)	101/207			80/131			15/20		
​ OR-1^a ^(95% CI)		1.03 (0.75–1.41)	0.867		1.29 (0.91–1.82)	0.155		1.58 (0.78–3.19)	0.201
​ OR-2^b ^(95% CI)		0.98 (0.71–1.37)	0.929		1.26 (0.88–1.82)	0.209		1.65 (0.7–1.95)	0.173
									
ALDH2 (*2:null/*2:null)	32/53			5/37			1/3		
​ OR-1^a ^(95% CI)		1.27 (0.78–2.06)	0.342		0.28 (0.11–0.73)	0.009		0.65 (0.07–6.33)	0.711
​ OR-2^b ^(95% CI)		1.16 (0.7–1.95)	0.56		0.26 (0.1–0.69)	0.007		0.61 (0.06–6.07)	0.675

^a^Logistic regression model controlling for matching factors only.

^b^Logistic regression model controlling for matching factors plus alcohol consumption, smoking status, body-mass index, regular exercise, family history of breast cancer, age at menarche, parity, hormone-replacement therapy and mode of referral to hospital.

There were 4 cases and 11 controls with unknown ADH1B and/or ALDH2 genotypes.

## DISCUSSION

In an evaluation of the impact of individual and combined *ADH1B* and *ALDH2* gene polymorphisms on the risk of breast cancer among a Japanese population, we found no significant effect modification by alcohol consumption.

To date, many epidemiological studies from Western countries have demonstrated that intake of alcoholic beverages is associated with female breast cancer risk, although the magnitude of this risk has been relatively modest.^2^^,^^3^ Recently, the IARC noted that there is sufficient evidence to classify alcohol as a carcinogen in the development of human female breast cancer.^4^ In contrast, among Japanese populations, the results have been inconsistent^28^^–^^30^ and a recent systematic review of a Japanese population concluded that epidemiological evidence for an association between alcohol drinking and breast cancer risk remains insufficient because of the small number and low methodological quality of the available studies.^31^ However, a recent large cohort study demonstrated that risk was significantly increased among Japanese women who consumed at least 15 g per day of alcohol.^30^ One possible explanation for this inconsistency is that the amount of alcohol consumed by Japanese women is lower than that consumed by white women.^32^ Therefore, studies of Japanese women might have been underpowered to detect the modest effect of alcohol intake. Another possibility is that different genetic backgrounds or different lifestyles in the Japanese population might reduce the effect of alcohol consumption on breast cancer risk.^33^ Whatever the case, confirmation of our findings, ideally by a large prospective cohort study, is needed.

Several studies have clearly demonstrated that the *ALDH2/ADH1B* genotypes, which would be expected to result in exposure to high acetaldehyde concentrations, are associated with an increased risk of some types of cancer among drinkers.^9^^–^^15^ Yokoyama et al found that individuals with ALDH2*1/*2 were at high risk for several types of cancer^10^ and that their risk for oropharyngolaryngeal and esophageal cancers was 11 to 24 times higher than for individuals with ALDH2*1/*1. They also found significantly increased risks for cancers of the stomach, colon, and lung, despite a relatively small number of subjects; however, they did not evaluate female breast cancer. Thus, if the association between alcohol drinking and breast cancer risk were real, even though modest, it would be intensified by the genetic modulation of *ALDH2* and/or *ADH1B,* which should make it more detectable. Therefore, the lack of gene–environment interaction between the effects of *ADH1B* and *ALDH2* gene polymorphisms and alcohol consumption on the risk of breast cancer in this study (Table 2 and 4) suggests that the carcinogenic effect of acetaldehyde for breast cancer, if present, must be weak, and that other mechanisms explain the carcinogenic effect of alcohol. This lack of association is consistent with a small Korean case–control study.^16^

The methodological background of this study warrants discussion. First, with regard to the control population, we used noncancer patients at the ACCH because our case subjects were from this population, and this guarantees internal validity. To account for differences in background between cases and controls, we adjusted for mode of referral to our hospital. Moreover, with regard to the external validity of our results, we previously showed that individuals selected randomly from our control population were similar to the general population from which they were drawn, in terms of the exposure of interest^24^; in addition, the genotype distributions of the *ALDH2* and *ADH1B* polymorphisms in our controls and the general population were consistent.^34^ Second, as with other case–control studies, this study may have suffered from recall bias. However, because the questionnaires were completed before diagnosis in our hospital the HERPACC system is less susceptible to this type of bias. A further potential source of bias was the medical background of the controls. Our previous study focusing on women demonstrated that this had only limited impact: more than 66% of noncancer outpatients at ACCH have no specific medical condition, and the remaining 34% have specific diseases such as benign tumors, non-neoplastic polyps or both (13.1%), mastitis (7.5%), gastrointestinal disease (4.1%), or benign gynecologic disease (4.1%),^35^ which indicates that any such bias would be limited. Finally, the study had sufficient power to assess the impact of *ALDH2* polymorphism on the risk of breast cancer. However, careful interpretation of results from the stratified and interaction analyses is necessary due to the limited number of subjects included in these analyses.

In conclusion, in this case–control study we found no significant impact of *ADH1B* and *ALDH2* gene polymorphisms on the risk of female breast cancer and no significant gene–environment interactions of the effects of alcohol consumption and polymorphisms in *ADH1B* and *ALDH2* on the risk of breast cancer, despite sufficient statistical power to observe such associations. These findings do not support the hypothesis that acetaldehyde is the main contributor to the carcinogenesis of alcohol-induced breast cancer.
